# Lysophosphatidic Acid Receptor 5 (LPA_5_) Antagonist AS2717638 Attenuates Allergic Asthma by Reducing Mast Cell Degranulation and Th2 Inflammation in Mice

**DOI:** 10.3390/ijms27167296

**Published:** 2026-08-15

**Authors:** Seung-Won Jeong, Dong-Soon Im

**Affiliations:** 1 Department of Fundamental Pharmaceutical Sciences, Graduate School, Kyung Hee University, Seoul 02447, Republic of Korea; 2025310813@khu.ac.kr; 2 Institute of Integrated Pharmaceutical Sciences, Kyung Hee University, Seoul 02447, Republic of Korea

**Keywords:** lysophosphatidic acid, LPA5, AS2717638, allergy, asthma, lpar5

## Abstract

Lysophosphatidic acid receptor 5 (LPA_5_, formerly designated GPR92) is a G protein-coupled receptor that recognizes lysophosphatidic acid. Previous studies have indicated that the absence of LPA_5_ confers protection against the development of neuropathic pain. Although LPA_5_ expression has been identified in mast cells, macrophages, and microglia, its specific contribution to allergic responses remains insufficiently characterized. In this study, we evaluated the therapeutic potential of LPA_5_ inhibition in allergic asthma. To this end, the effects of the selective LPA_5_ antagonist 6,7-dimethoxy-2-(5-methyl-1,2-benzisoxazol-3-yl)-4-(1-piperidinylcarbonyl)-1(2H)-isoquinolinone (AS2717638) were examined using both an in vitro mast cell degranulation model and an in vivo ovalbumin-induced asthma model in BALB/c mice. In RBL-2H3 cells expressing the *lpar5* gene, AS2717638 treatment attenuated antigen-induced degranulation. In vivo, administration of AS2717638 reduced airway resistance triggered by ovalbumin exposure. Furthermore, treatment with AS2717638 led to a decrease in eosinophil and lymphocyte infiltration, as well as reduced levels of inflammatory cytokines in bronchoalveolar lavage fluid. Histopathological analysis additionally demonstrated diminished pulmonary inflammation following AS2717638 administration, accompanied by significant reductions in cytokine expression within lung tissue. Collectively, these findings suggest that pharmacological inhibition of LPA_5_ using AS2717638 mitigates key features of allergic asthma, supporting LPA_5_ as a potential therapeutic target for this condition.

## 1. Introduction

Asthma, a chronic inflammatory disorder of the airways, has shown a marked increase in global prevalence and is now widely recognized as a common disease [1]. Among experimental approaches, allergen-driven models of asthma are the most extensively utilized for investigating disease mechanisms and therapeutic strategies [2]. Current standard treatments include long-acting β-adrenergic agonists for bronchodilation, inhaled corticosteroids for inflammation control, and leukotriene D_4_ receptor antagonists [3]. Although biologic agents targeting immunological mediators such as IgE, interleukin (IL)-5, IL-4, and IL-13 have demonstrated clinical benefit, a subset of patients with severe asthma remains inadequately responsive to these therapies, highlighting the need for alternative treatment options [3]. In this context, lipid-sensing G protein-coupled receptors have emerged as potential therapeutic targets due to their roles in regulating inflammatory and immune responses. Previous efforts have explored receptors such as sphingosine-1-phosphate receptors (S1P_2_ and S1P_4_), free fatty acid receptors (FFA2, FFA3, and FFA4/GPR120), GPR119, and the G protein-coupled estrogen receptor (GPER) [4,5,6,7,8]. Building on these approaches, the present study focuses on lysophosphatidic acid receptor 5 (LPA_5_, formerly known as GPR92) to evaluate its potential involvement in allergic asthma [9,10].

Lysophosphatidic acid (LPA) is a bioactive extracellular lipid mediator that signals through a family of G protein-coupled receptors, designated LPA_1_–LPA_6_ [11]. Elevated concentrations of LPA have been detected in bronchoalveolar lavage fluid (BALF) following allergen exposure, with levels markedly increased approximately 18 h after challenge in asthmatic lungs [12]. Similarly, BALF obtained from mice sensitized and challenged with *Schistosoma mansoni* eggs exhibits an approximately 2.8-fold increase in LPA levels [13]. Accumulating evidence indicates that endogenous LPA in the lung functions as a key regulator of pro-inflammatory and profibrotic processes in various murine models of pulmonary disease, including asthma [12,13], pulmonary fibrosis [14], and acute lung injury [15]. Mechanistic studies have demonstrated that LPA_1_ plays a critical role in the pathogenesis of pulmonary fibrosis, leading to the development of therapeutic candidates targeting idiopathic pulmonary fibrosis [14,16]. LPA_2_ has been shown to facilitate transforming growth factor-β1 (TGF-β1) production via Gαq-dependent signaling in lung epithelial cells [17], and it has been implicated in multiple aspects of allergic airway inflammation and asthma pathogenesis [13,18,19]. In contrast, LPA_5_, which is structurally related to purinergic GPCRs, remains largely unexplored in the context of allergic asthma, despite its expression in multiple tissues, including the lung, spleen, thymus, stomach, small intestine, and liver [9,10]. Notably, LPA_5_ is also expressed in immune cells such as mast cells, macrophages, and microglia [9,10,14,20]. Given this expression profile and the established roles of LPA signaling in inflammatory responses, we investigated whether pharmacological inhibition of LPA_5_ using a selective antagonist, 6,7-dimethoxy-2-(5-methyl-1,2-benzisoxazol-3-yl)-4-(1-piperidinylcarbonyl)-1(2H)-isoquinolinone (AS2717638), was examined using both an in vitro mast cell degranulation model and an in vivo ovalbumin-induced asthma model in BALB/c mice [14,21,22].

## 2. Results

### 2.1. Single-Cell RNA-Sequencing Data Showed Strong Expression Levels of LPAR5 in Immune Cells of Human Lungs

We searched mRNA expression levels of *LPAR5* in various cell types of human lungs. Data by single-cell RNA-sequencing of human lungs are shown in Supplemental Figure S1, which is retrieved from the website of Human Protein Atlas (https://v22.proteinatlas.org/ENSG00000184574-LPAR5, accessed on 24 April 2026). Data suggested strong expression levels of *LPAR5* mRNA mainly in granulocytes (c-14), T cells (c-16 and c-10), B cells (c-20), and macrophages (c-12 and c-22), but not in smooth muscle cells, club cells, alveolar type 1 and 2 cells, respiratory ciliated cells, and endothelial cells (Supplemental Figure S1), confirming the previous data in immune cells and human mast cells [9,10,22].

### 2.2. AS2717638 Inhibited Antigen-Induced Degranulation in the RBL-2H3 Cells

**Figure 1:**
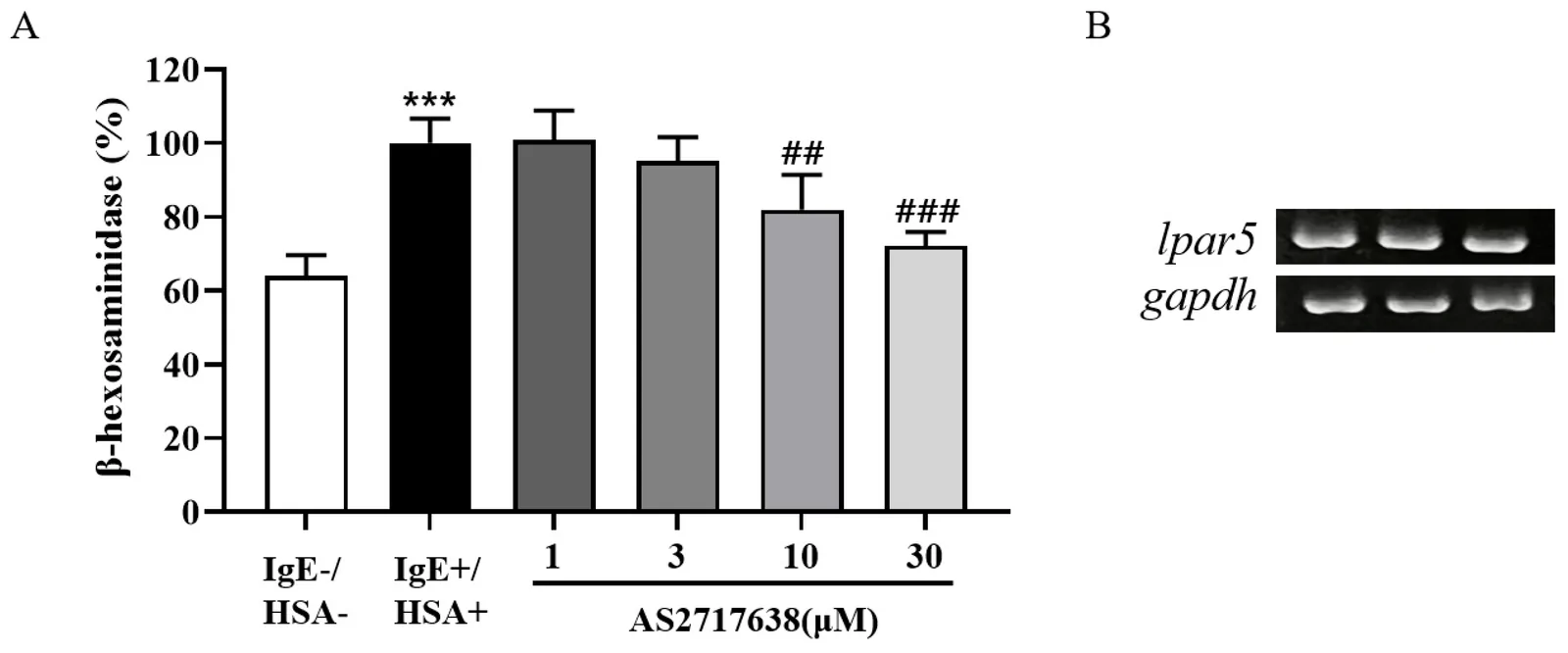
AS2717638 inhibited antigen-induced degranulation in RBL-2H3 cells. (**A**) RBL-2H3 cells were sensitized with anti-dinitrophenyl (DNP) IgE for 18 h, followed by stimulation with DNP-conjugated human serum albumin (HSA). AS2717638 was administered at the indicated concentrations 30 min prior to antigen challenge. Conditions lacking both IgE and HSA were used to represent basal degranulation, whereas cells treated with both IgE and HSA served as the positive control for antigen-induced degranulation. The results are presented as the means ± SEs of three independent experiments. *** *p* < 0.001 vs. the HSA-untreated group. ^##^ *p* < 0.01, ^###^ *p* < 0.001 vs. the HSA-treated group. (**B**) RT-PCR results of *lpar5* and *gapdh* in RBL-2H3 cells. As shown, RNAs were extracted from RBL-2H3 cells three times independently.

Mast cells are key effector cells in the pathophysiology of asthma [23]. Antigen exposure induces cross-linking of immunoglobulin E (IgE) bound to the mast cell surface, triggering cellular degranulation. This process leads to the release of various mediators involved in allergic inflammation, including histamine, leukotrienes, and prostaglandins [24]. To evaluate degranulation responses, RBL-2H3 rat basophilic leukemia cells were employed as an in vitro model [25]. Antigen stimulation markedly increased β-hexosaminidase activity in the culture medium of RBL-2H3 cells (Figure 1A). Treatment with AS2717638 significantly reduced β-hexosaminidase release at concentrations of 10 and 30 μM (Figure 1A), indicating an inhibitory effect on mast cell degranulation.

### 2.3. AS2717638 Inhibited the Airway Hyperresponsiveness (AHR) During Ovalbumin (OVA)-Induced Asthma

**Figure 2:**
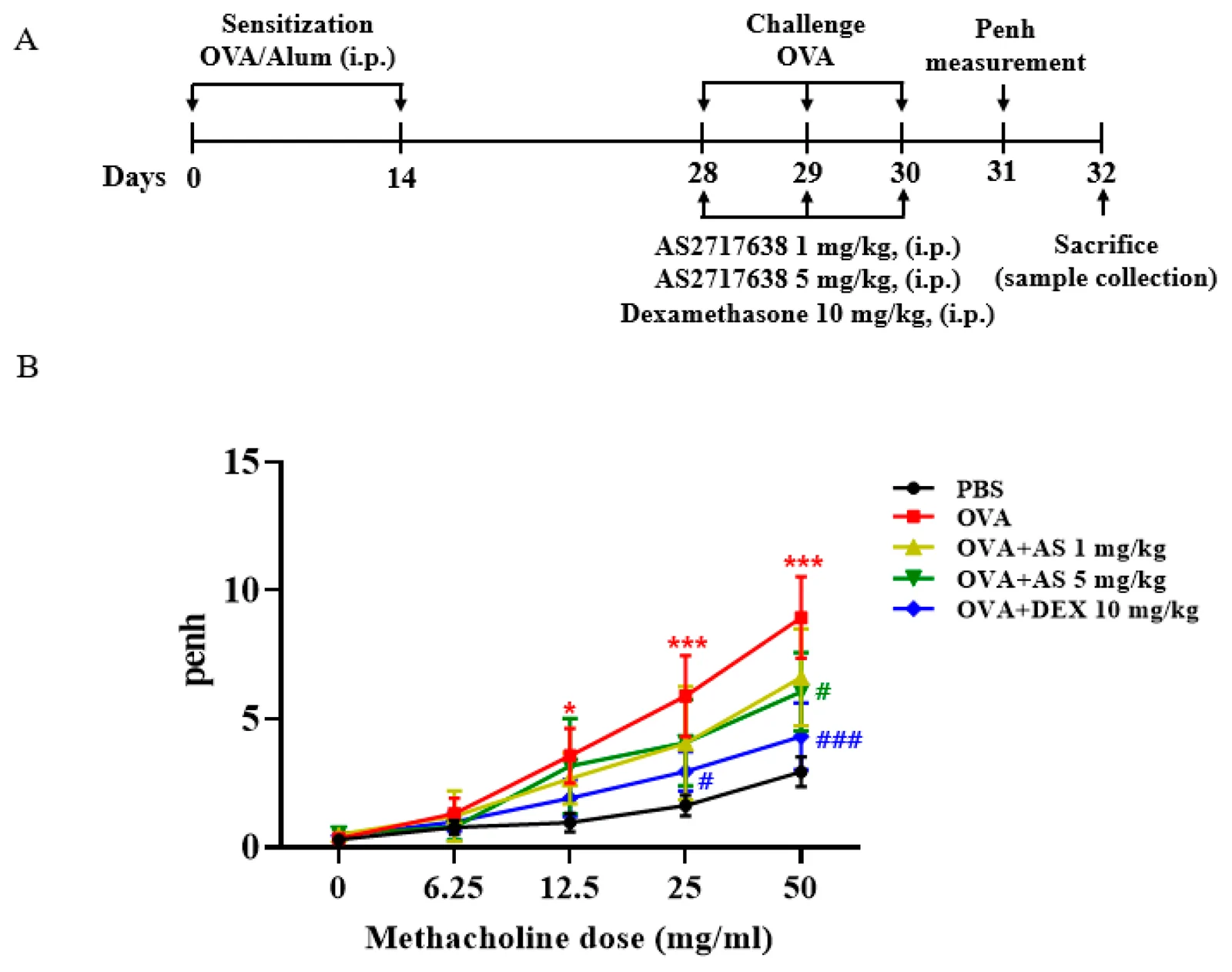
AS2717638 inhibits OVA-induced airway hyperresponsiveness (AHR). (**A**) Schematic representation of the experimental design for the ovalbumin (OVA)-induced allergic asthma model. (**B**) Airway hyperresponsiveness (AHR) was evaluated following AS2717638 treatment using non-invasive whole-body plethysmography. Values are presented as the mean ± standard error of the mean (SEM) of six independent experiments. * *p* < 0.05, *** *p* < 0.001 vs. PBS group, ^#^ *p* < 0.05, ^###^ *p* < 0.001 vs. OVA group. OVA, ovalbumin; PBS, phosphate-buffered saline.

Allergic asthma was established in BALB/c mice through ovalbumin (OVA) sensitization and subsequent airway challenges, as described in the Methods. The impact of AS2717638 on allergic immune responses was then evaluated. The selected doses of AS2717638 (1 and 5 mg/kg) were based on prior in vivo studies conducted in rodent models [21,26,27]. The compound was administered via intraperitoneal injection, a route commonly employed in similar experimental settings [21,26,27]. Airway hyperresponsiveness (AHR) was assessed by measuring enhanced pause (Penh) values. OVA-sensitized mice exhibited a marked increase in Penh compared with control animals at methacholine concentrations ranging from 12.5 to 50 mg/mL, confirming successful induction of the asthma phenotype. Administration of AS2717638 attenuated this increase in Penh (Figure 2), with a statistically significant reduction observed at the 5 mg/kg dose in response to 50 mg/mL methacholine (Figure 2). In comparison, the reference compound dexamethasone demonstrated greater efficacy and potency in reducing AHR (Figure 2).

### 2.4. AS2717638 Suppressed the Immune Cell Counts in the Bronchoalveolar Lavage Fluid (BALF)

**Figure 3:**
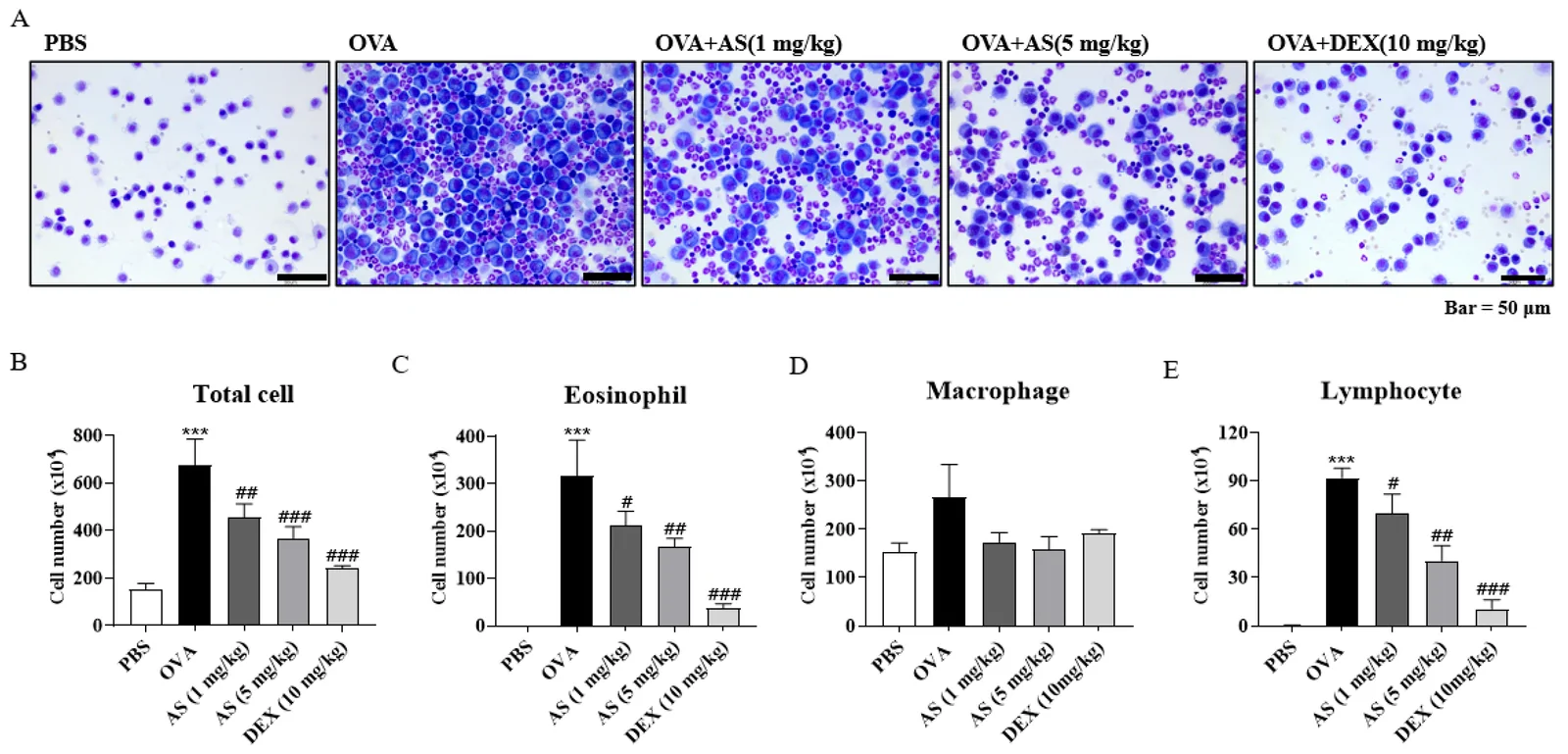
AS2717638 inhibited OVA-induced immune cell accumulation in BALF. (**A**) Mice were sensitized by intraperitoneal administration of ovalbumin (OVA) on days 0 and 14, followed by aerosolized OVA challenges on days 28, 29, and 30. AS2717638 was administered intraperitoneally at doses of 1 or 5 mg/kg, 30 min prior to each OVA challenge. Bronchoalveolar lavage fluid (BALF) cells were stained with May–Grünwald solution and subsequently quantified. (**B**) Total cell counts, (**C**) eosinophil counts, (**D**) macrophage counts, and (**E**) lymphocyte counts in BALF. The results are presented as the mean ± SE cell count values (*n* = 6). *** *p* < 0.001 vs. the PBS-treated group, ^#^ *p* < 0.05, ^##^ *p* < 0.01, ^###^ *p* < 0.001 vs. the OVA-treated group.

Immune cell populations in bronchoalveolar lavage fluid (BALF) were subsequently analyzed. The OVA-induced asthma group demonstrated a substantial increase in total immune cell counts compared with the control group (Figure 3A), whereas treatment with AS2717638 (1 or 5 mg/kg) attenuated this elevation (Figure 3A). As shown in Figure 3B, OVA challenge markedly increased the total number of cells in BALF to 439.4% relative to controls. This increase was significantly reduced by AS2717638 treatment, with decreases of 32.5% and 45.9% observed at doses of 1 and 5 mg/kg, respectively (Figure 3B). Similarly, OVA exposure led to a pronounced rise in eosinophil counts, which was significantly suppressed by AS2717638 administration by 33.0% and 47.4% at 1 and 5 mg/kg, respectively (Figure 3C). In contrast, macrophage numbers were not significantly affected by either OVA challenge or AS2717638 treatment (Figure 3D). Lymphocyte counts were significantly elevated following OVA exposure, and this increase was significantly reduced by AS2717638 treatment by 24.1% and 56.0% at 1 and 5 mg/kg, respectively (Figure 3E).

### 2.5. AS2717638 Inhibited the Immune Responses in the Bronchoalveolar Lavage Fluid (BALF)

**Figure 4:**
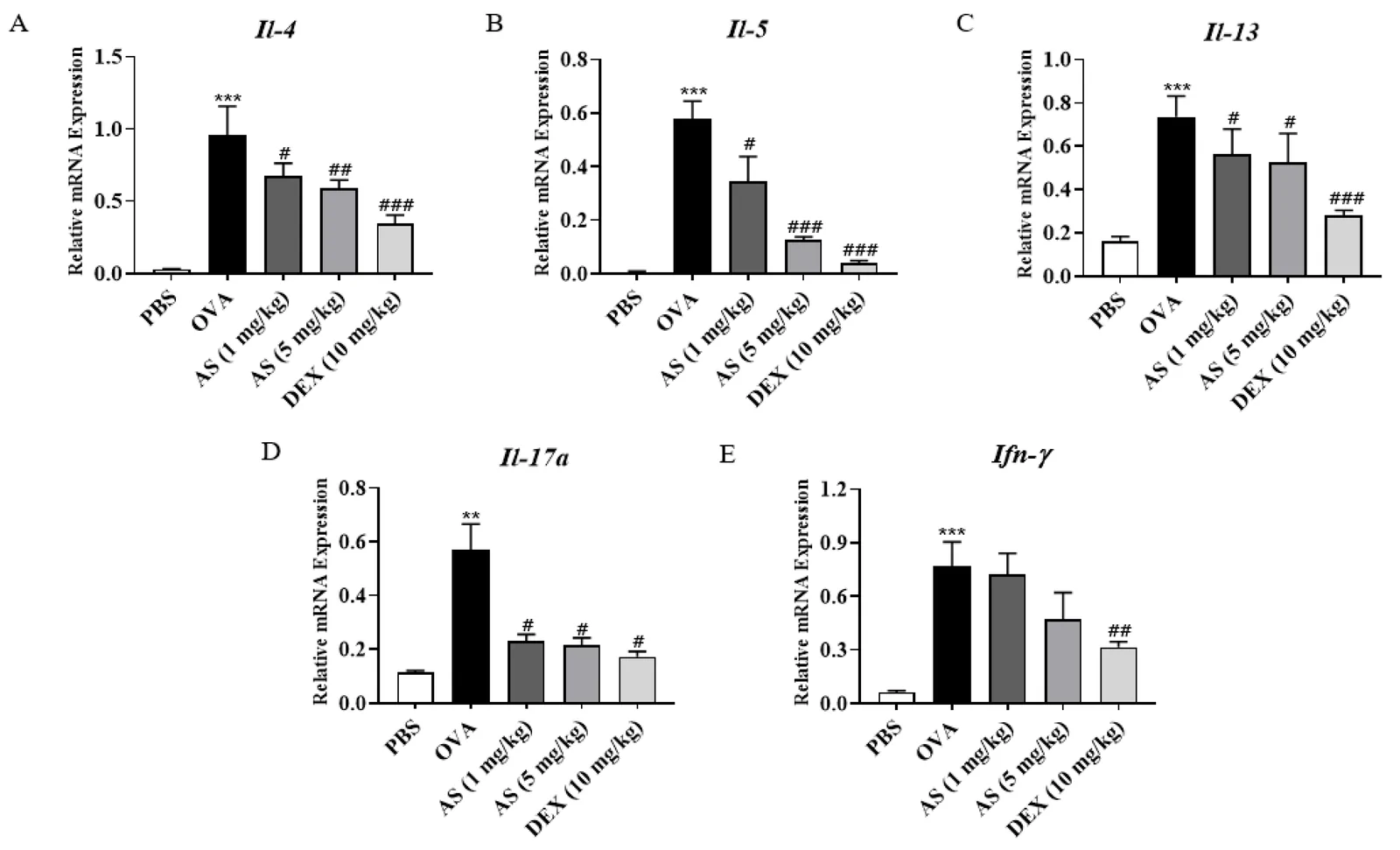
AS2717638 inhibited the mRNA expression levels of Th2/Th17/Th1 cytokines in BALF-associated cells. (**A**–**E**) mRNA expression levels of Th2 cytokines, including *Il-4* (**A**), *Il-5* (**B**), and *Il-13* (**C**), as well as the Th17 cytokine *Il-17A* (**D**) and the Th1 cytokine *Ifn-γ* (**E**), were analyzed in BALF-associated cells. Cytokine expression levels were normalized to *Gapdh* mRNA and are presented as relative expression ratios. The results are presented as the mean ± SE values of qPCR (*n* = 6). ** *p* < 0.01, *** *p* < 0.001 vs. the PBS-treated group, ^#^ *p* < 0.05, ^##^ *p* < 0.01, ^###^ *p* < 0.001 vs. the OVA-treated group.

Allergic asthma is predominantly mediated by T helper 2 (Th2)-associated cytokines, including interleukin (IL)-4, IL-5, and IL-13 [28], while Th1- and Th17-related cytokines are known to contribute to the progression of chronic disease states [29]. To evaluate these responses, the mRNA expression levels of representative Th2, Th1, and Th17 cytokines in BALF cells were quantified using real-time reverse transcription PCR (qRT-PCR) (Figure 4A–E).

Compared with the control group, mice subjected to OVA challenge exhibited significant upregulation of IL-4, IL-5, IL-13, IL-17A, and interferon (IFN)-γ transcripts. Treatment with AS2717638 markedly reduced the elevated expression of IL-4, IL-5, IL-13, and IL-17A, whereas IFN-γ levels were not significantly affected (Figure 4A–E).

### 2.6. AS2717638 Inhibited the Inflammatory Responses in the Lung Tissues

**Figure 5:**
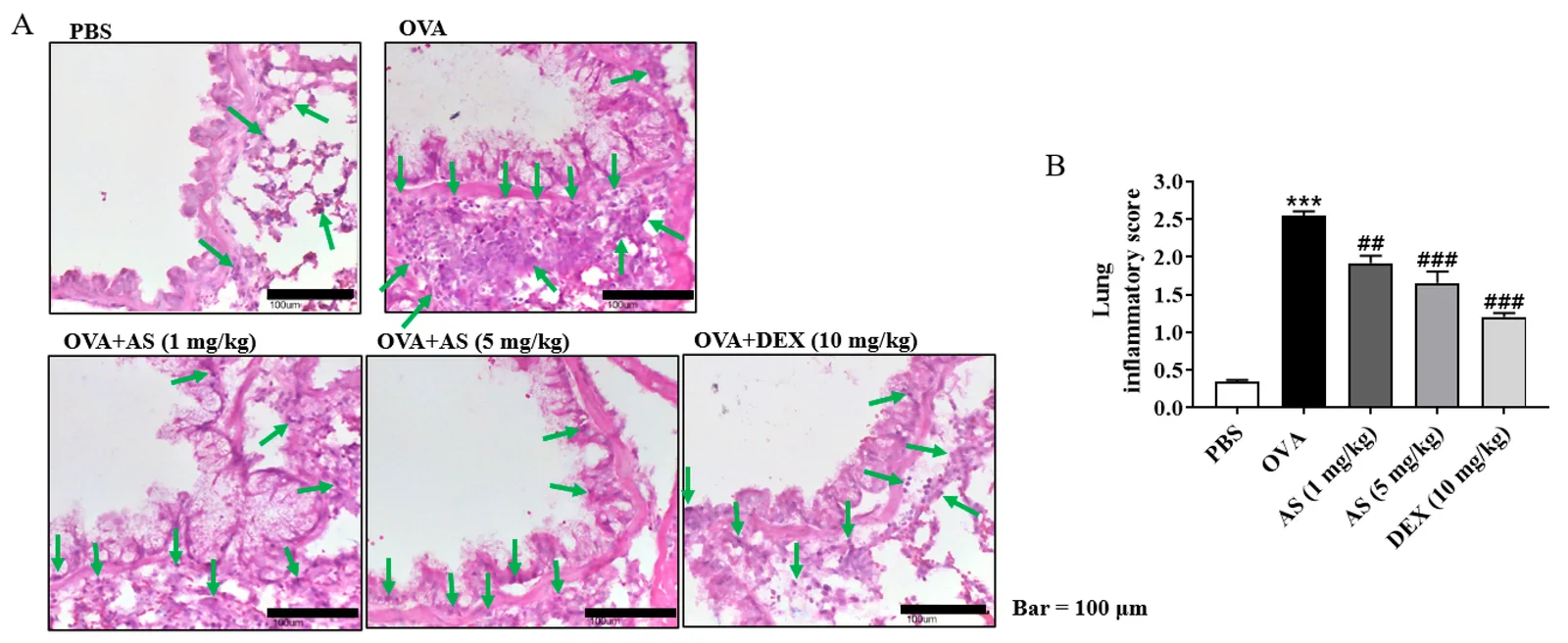
AS2717638 protected against airway inflammation. (**A**) Representative H&E-stained lung sections from the PBS control group, OVA-challenged group, and OVA groups treated with AS2717638 (1 or 5 mg/kg) are shown. Immune cells appear as small, dark blue-stained cells surrounding the bronchioles. Minimal infiltration of immune cells was observed in the PBS group, whereas marked peribronchiolar accumulation was evident in the OVA group (indicated by green arrows). This infiltration of immune cells was noticeably reduced in the AS2717638-treated groups compared with the OVA group. (**B**) The extent of lung inflammation was assessed semi-quantitatively, and histopathological scores were assigned according to the criteria described in the Materials and Methods section. Values represent the means ± SEs (*n* = 6). *** *p* < 0.001 vs. the PBS-treated group, ^##^ *p* < 0.01, ^###^ *p* < 0.001 vs. the OVA-treated group.

Hematoxylin and eosin (H&E) staining demonstrated the accumulation of inflammatory cells, appearing as small, dark blue-stained structures surrounding the bronchioles, consistent with infiltration of immune cells (Figure 5A) [30,31]. Minimal presence of immune cells was observed in the control group, whereas mice in the OVA-challenged group exhibited pronounced peribronchiolar infiltration of immune cells (Figure 5A). Administration of AS2717638 (1 or 5 mg/kg) markedly attenuated this OVA-induced increase in the accumulation of immune cells (Figure 5A). The immune cells are supposed to be eosinophils because the major immune cells recruited in the model were eosinophils as shown in Figure 3 [30,31]. Pulmonary inflammation was further assessed using a semi-quantitative scoring system ranging from 0 to 3. The OVA group displayed an average inflammation score of 2.6, which was significantly reduced following treatment with AS2717638 (Figure 5B).

### 2.7. AS2717638 Inhibited Mucin Secretion in the Lung Tissues

**Figure 6:**
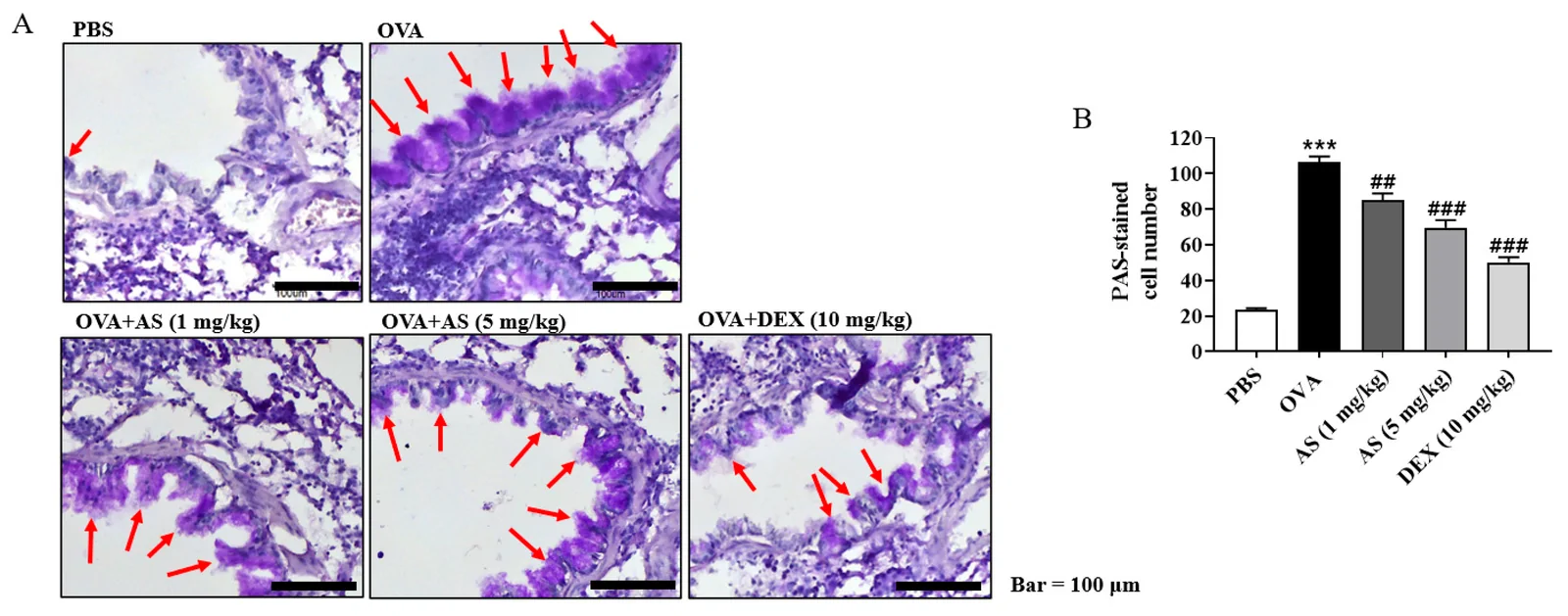
AS2717638 protected against mucin production. (**A**) Representative PAS/hematoxylin-stained lung sections from the PBS control group, OVA-challenged group, and OVA groups treated with AS2717638 (1 or 5 mg/kg) are presented. In PAS staining, mucin appears as a purple coloration. Compared with the PBS group, the OVA group exhibited more intense and thicker purple staining surrounding the bronchioles, indicating increased mucus accumulation. (**B**) Mucus production was quantified by counting PAS-positive cells (indicated by red arrows) per millimeter of bronchiole (*n* = 6 per group). *** *p* < 0.001 vs. the PBS-treated group, ^##^ *p* < 0.01, ^###^ *p* < 0.001 vs. the OVA-treated group.

Periodic acid–Schiff (PAS) staining revealed mucin-containing or mucin-secreting goblet cells as intensely violet-stained structures (Figure 6A). In the OVA-challenged group, goblet cell hyperplasia was prominently observed around the bronchioles, whereas treatment with AS2717638 reduced the intensity of PAS staining (Figure 6A). Mucin-producing cells within the bronchioles were quantified in a quantitative manner (Figure 6B). The control group exhibited minimal numbers of PAS-positive cells, while the OVA group showed a marked increase, reaching approximately 106.6 PAS-positive cells per millimeter of bronchiole. Administration of AS2717638 significantly decreased the number of PAS-positive cells (Figure 6B), indicating suppression of mucus production.

### 2.8. AS2717638 Inhibited Immune Responses in Lung Tissues

In addition, the mRNA expression levels of IL-4, IL-5, IL-13, IL-17A, and IFN-γ were examined in lung tissues (Supplemental Figure S2A–E). All measured cytokines were markedly upregulated in the OVA-challenged group, indicating enhanced Th2-, Th1-, and Th17-associated responses (Supplemental Figure S2A–E). Treatment with AS2717638 attenuated these OVA-induced increases in cytokine expression in 5 mg/kg AS2717638-treated group, but not in 1 mg/kg AS2717638-treated group except IL-5 (Supplemental Figure S2A–E).

### 2.9. AS2717638 Inhibited OVA-Induced Increases in BALF IL-13 and IL-4 Levels and Serum IgE Levels

**Figure 7:**
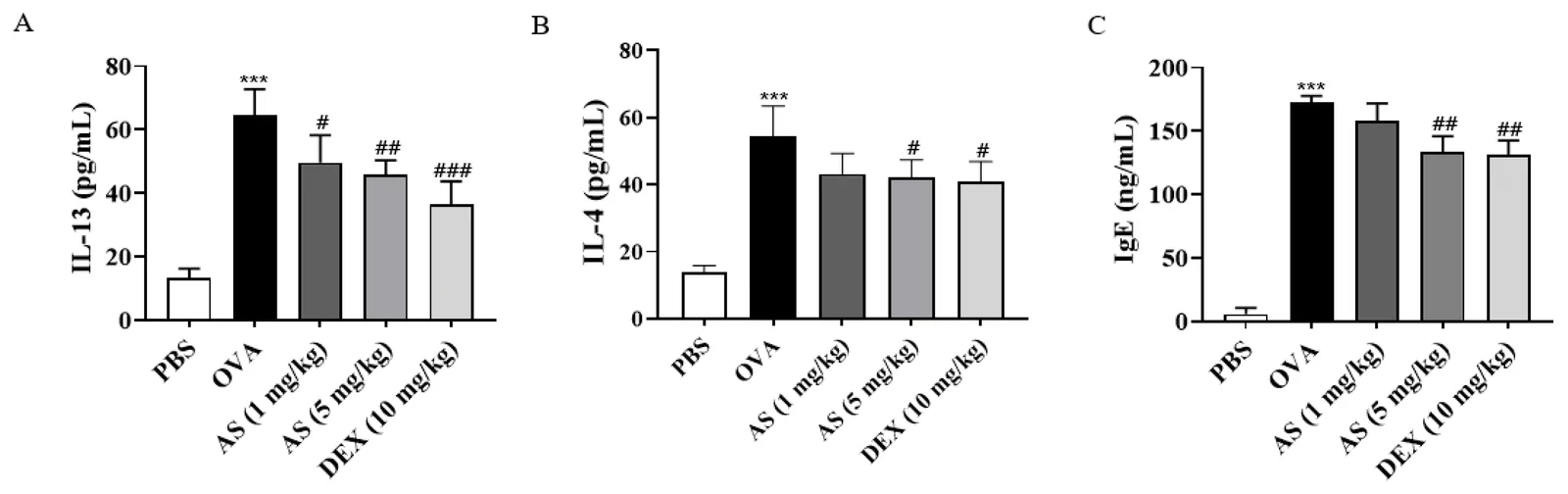
AS2717638 inhibits OVA-induced IL-13 and IL-4 levels in BALF and IgE levels in the serum. Enzyme-linked immunosorbent assay (ELISA) was performed to quantify protein levels of (**A**) IL-13 and (**B**) IL-4 in bronchoalveolar lavage fluid (BALF), and (**C**) serum IgE in OVA-induced asthmatic mice. Results are presented as the mean ± standard error of the mean (SEM) (*n* = 6). *** *p* < 0.001 vs. the PBS-treated group, ^#^ *p* < 0.05, ^##^ *p* < 0.01, ^###^ *p* < 0.001 vs. the OVA-treated group. BALF, bronchoalveolar lavage fluid; IL-13, interleukin-13; PBS, phosphate-buffered saline; OVA, ovalbumin.

IL-13 and IL-4, representative Th2 cytokines, are central mediators in the development of allergic asthma. The protein concentrations of IL-13 and IL-4 in BALF were quantified by ELISA. IL-13 levels were significantly elevated in the OVA-treated group, and this increase was significantly reduced following administration of AS2717638 (Figure 7A). Similarly, IL-4 levels were significantly increased in OVA-treated mice, and this increase was attenuated by AS2717638 treatment, with a statistically significant reduction observed at the 5 mg/kg dose (Figure 7B).

To further evaluate systemic immune responses, serum IgE levels were measured. IgE concentrations were increased in mice treated with OVA (Figure 7C). AS2717638 treatment significantly reduced the OVA-induced increase in serum IgE at the 5 mg/kg dose, whereas DEX also effectively suppressed serum IgE levels (Figure 7C).

## 3. Discussion

In the present study, we demonstrated that pharmacological inhibition of LPA_5_ using AS2717638 attenuates allergic immune responses in both RBL-2H3 mast cells and an OVA-induced murine model of asthma. AS2717638 treatment significantly reduced inflammatory responses in BALF and lung tissues, which was accompanied by a decrease in airway hyperresponsiveness, as reflected by reduced Penh values. These findings are supported by a recent publication on pediatric asthma as follows: treatment of LPA_5_ antagonist reduced inflammatory cell infiltration in the lung tissues of OVA-challenged neonatal mice [32]. Gene expression of *LPAR5* was higher in pediatric patients with asthma compared with healthy controls, implying the significance of LPA_5_ in asthmatic pathogenesis [32]. Li et al. elucidated that the significantly elevated proportion of F4/80-positive macrophages in pediatric asthma and its reduction by treatment of LPA_5_ antagonist [32], while we were not able to find significant change in macrophage counts (Figure 3). Considering the wide expression of *LPAR5* in immune cells in the human lung tissues (Supplemental Figure S1), not limited to macrophages [32], functional roles of LPA_5_ in granulocytes like mast cells and basophils may contribute to allergic asthma phenotypes. While AS2717638 significantly reduces asthma-related endpoints, the direct involvement of Th2, Th1, or Th17 cell inhibition remains to be fully elucidated. Also, a limitation of this study is that the qRT-PCR analysis was performed on total BALF cells. Therefore, the reduced cytokine transcript levels may partly reflect alterations in the overall cellular composition of the BALF rather than a strict decrease in expression levels per individual cell.

Previous studies have shown that genetic deletion of LPA_5_ confers protection against neuropathic pain [33], and AS2717638 has been reported to exert analgesic effects in rodent models of neuropathic and inflammatory pain. In microglial systems, LPA_5_ signaling has been implicated in the production of pro-inflammatory mediators, including IL-6, TNF-α, IL-1β, CXCL10, CXCL2, and CCL5 [26], while in vivo inhibition of LPA_5_ reduces endotoxin-induced neuroinflammatory markers such as TLR4, Iba1, GFAP, and COX-2 [27]. Furthermore, LPA_5_-dependent signaling has been associated with inflammatory skin disorders, including psoriasis, through mechanisms involving macrophage NLRP3 activation [34]. Consistent with these findings, the present data extend the pro-inflammatory role of LPA_5_ to allergic airway disease, demonstrating its involvement in Th2-driven immune responses, in addition to previously described roles in Th1- and Th17-mediated inflammation. LPA_5_ has also been implicated in M1-like polarization of microglia, neuroinflammatory damage following cerebral ischemia, and mast cell activation, where it mediates LPA-induced chemokine release [22,35,36,37]. Moreover, its contribution to neuroinflammatory conditions such as multiple sclerosis further supports a broad role for LPA_5_ in inflammation-associated pathologies [22,37,38].

In summary, our findings indicate that the selective LPA_5_ antagonist AS2717638 attenuates key features of allergic asthma by partly inhibiting mast cell degranulation and reducing the production of pro-inflammatory cytokines associated with Th2, Th1, and Th17 responses. These results suggest that endogenously generated LPA may promote allergic inflammation through LPA_5_ signaling and highlight LPA_5_ as a promising therapeutic target for the treatment of allergic diseases, including asthma.

## 4. Materials and Methods

### 4.1. Materials

AS2717638 was obtained from MedChemExpress (Monmouth Junction, NJ, USA). Ovalbumin and aluminum hydroxide were sourced from Sigma-Aldrich (St. Louis, MO, USA).

### 4.2. Cell Culture

Rat basophilic leukemia RBL-2H3 cells were obtained from the American Type Culture Collection (ATCC; Manassas, VA, USA). The cells were maintained at 37 °C in a humidified incubator with 5% CO_2_ and cultured in high-glucose Dulbecco’s modified Eagle’s medium (DMEM) supplemented with 10% (*v*/*v*) heat-inactivated fetal bovine serum, 2 mM glutamine, 100 U/mL penicillin, 1 mM sodium pyruvate, and 50 μg/mL streptomycin [7].

### 4.3. Assessment of Degranulation

Degranulation of RBL-2H3 cells was evaluated by quantifying β-hexosaminidase activity released into the culture medium. AS2717638 was initially prepared as a 100 mM stock solution in dimethyl sulfoxide (DMSO) and subsequently diluted in PIPES buffer immediately prior to use. Degranulation was induced using a monoclonal anti-dinitrophenyl (DNP) mouse immunoglobulin E in combination with dinitrophenyl-conjugated human albumin [7].

### 4.4. BALB/c Mice

All animal procedures were approved by the Institutional Animal Care Committee of Kyung Hee University (KHSASP-26-331). Five-week-old BALB/c mice were obtained from Daehan Biolink (Eumseong-gun, Chungcheongbuk-do, Republic of Korea) and housed in pairs under controlled environmental conditions (22–24 °C, 60 ± 5% relative humidity). Animals were provided with standard laboratory chow and water *ad libitum*. At the end of the experiment, mice were humanely euthanized in a chamber by gradual displacement of air with carbon dioxide (CO_2_) at a flow rate of 30% to 70% of the chamber volume per minute, in accordance with the AVMA Guidelines for the Euthanasia of Animals.

### 4.5. Ovalbumin (OVA)-Induced Asthma Induction in BALB/c Mice

Six-week-old female BALB/c mice were randomly assigned to the following five experimental groups (*n* = 6 per group): a PBS-treated control group, an ovalbumin (OVA)-induced asthma group, OVA-induced groups receiving AS2717638 (1 or 5 mg/kg), and an OVA-induced group receiving dexamethasone (10 mg/kg). AS2717638 was prepared as a stock solution (10 mg/mL) in dimethyl sulfoxide (DMSO) and diluted in corn oil immediately prior to administration based on individual body weight (e.g., 2.2 μL stock for a 22 g mouse, adjusted to a final volume of 50 μL), followed by intraperitoneal injection. Sensitization was achieved by intraperitoneal administration of 50 μg OVA combined with 1 mg aluminum hydroxide on days 0 and 14 (Figure 2A). Airway challenge was conducted on days 28–30 by exposure to nebulized 1% OVA or PBS for 30 min using an ultrasonic nebulizer (Figure 2A). AS2717638 was administered intraperitoneally 30 min prior to challenge (days 28–30) (Figure 2A). Bronchoalveolar lavage fluid (BALF) was collected on day 32, and cellular composition was analyzed following staining procedures [7]. No animals or data points were excluded from the final analysis. Due to the nature of the interventions, the researchers conducting the experiment were aware of the group allocation. However, the outcome assessment and data analysis were performed by independent researchers who were blinded to the treatment groups. The primary outcome measure was the change in immune cells in the BALF, which was used to determine the sample size a priori. Secondary outcome measures included the expression levels of inflammatory cytokines (IL-4, IFN-γ) measured by qPCR. From each euthanized mouse, BALF was collected first as described above, and the cellular pellet was processed for RNA isolation. Subsequently, the whole lung tissue was harvested from the same animal, snap-frozen in liquid nitrogen, and homogenized for tissue qRT-PCR analysis, ensuring paired biological replication (*n* = 6 mice per group).

### 4.6. Cell Counting and Analysis in BALF

Bronchoalveolar lavage fluid (BALF) was collected by instilling 0.5 mL of ice-cold phosphate-buffered saline (PBS) into the trachea via a cannulate catheter. This procedure was repeated twice, and recovered fluid was centrifuged 2000 rpm for 10 min at 4 °C to isolate cells. Cells obtained from BALF were cytospun onto glass slides using a Cellspin^®^ centrifuge (Hanil Electric, Seoul, Republic of Korea) and subsequently fixed in methanol for 30 s. The slides were sequentially stained with May–Grünwald solution for 8 min, followed by Giemsa staining for 12 min. Differential cell counts were performed independently by two investigators based on morphological characteristics, including cell size, staining intensity, and granularity. Macrophages were identified as large cells with blue staining, eosinophils as mid-sized cells containing purple cytoplasmic granules, and lymphocytes as smaller cells exhibiting intense blue staining.

### 4.7. Measuring Airway Hyperresponsiveness (AHR) to Methacholine

Airway hyperresponsiveness (AHR) was assessed one day after the final ovalbumin (OVA) challenge using a non-invasive lung function measurement system (Model PLY-UNR-MS2; EMKA Technologies, Paris, France). Mice were placed in a barometric plethysmography chamber, and baseline measurements were recorded for 3 min. Enhanced pause (Penh) values were then calculated in accordance with the manufacturer’s instructions. AHR was expressed as the percentage increase in Penh in response to escalating concentrations of methacholine (0, 6.25, 12.5, 25, and 50 mg/mL) [7].

### 4.8. Histological Examination of the Lung

Lung tissues were fixed in 10% neutral buffered formalin for 24 h. Following fixation, tissues were dehydrated through a graded series of ethanol, cleared in xylene, and embedded in O.C.T. solution. Sagittal tissue sections were cut at a thickness of 8 μm using a microtome. Sections were then stained with hematoxylin and eosin (H&E) and Periodic acid–Schiff (PAS) to evaluate airway inflammation and goblet cell hyperplasia, respectively. For quantification, 6 animals were evaluated per experimental group. From each animal, 2 non-consecutive lung sections were examined. For each section, 10 randomly selected microscopic fields surrounding bronchioles were evaluated. Microscopic fields were selected based on the presence of intact, cross-sectioned terminal bronchioles, avoiding large blood vessels or artifactual tissue damage. Scoring and cell quantification were performed at 200× magnification using a light microscope. Airway inflammation was scored on a scale of 0 to 3 based on the following complete definitions: score 0: normal lung morphology with no inflammatory cell infiltration. Score 1: mild inflammation with a few inflammatory cells forming a discontinuous layer around the peribronchial/perivascular spaces. Score 2: Moderate inflammation with a continuous, 1-to-3-cell-thick layer of inflammatory cells surrounding the airways. Score 3: Severe, dense inflammation with a thick layer (>3 cells deep) of inflammatory cells forming prominent cuffs around the airways, accompanied by luminal cell debris. To eliminate bias, all histological scoring and cell counting were performed by an independent observer who was strictly blinded to the experimental group allocations [5]. PAS-positive mucin-secreting cells surrounding the bronchioles were quantified in two sections per mouse. Mucus production was expressed as the number of PAS-positive cells per millimeter of bronchiole, determined by measuring the length of the bronchial basement membrane using ImageJ software v1.54t (National Institutes of Health) [7].

### 4.9. Quantitative Real-Time PCR

Total RNA was extracted from BALF-associated cells and lung tissues using TRIzol^®^ reagent (Invitrogen, Waltham, MA, USA). The isolated RNA was reverse-transcribed into complementary DNA (cDNA) using Moloney murine leukemia virus (MMLV) reverse transcriptase (Promega, Madison, WI, USA). Quantitative real-time PCR (qRT-PCR) was subsequently performed using Thunderbird Next SYBR qPCR Mix on a CFX Connect Real-Time PCR system (Bio-Rad, Hercules, CA, USA). Specific primers are listed in Table 1. The PCR program consisted of a cycle at 95 °C for 4 min, 40 cycles at 95 °C for 30 s and 57 °C for 30 s, and the last at 95 °C for 30 s. Results were normalized to the level of *Gapdh* gene expression [7].

### 4.10. Measurement of the Levels of Total Serum IgE and BALF IL-13 and IL-4

Serum IgE concentrations in mice were measured using enzyme-linked immunosorbent assay (ELISA) kits (eBioscience, San Diego, CA, USA). Levels of IL-4 and IL-13 in bronchoalveolar lavage fluid (BALF) were also quantified by ELISA. Cytokine-specific capture and biotinylated detection antibodies were purchased from eBioscience (IL-4: Cat. Nos. 14-7041-68 and 33-7042-68C; IL-13: Cat. Nos. 14-7043-68 and 33-7135-68B). Signal detection was performed using avidin–horseradish peroxidase, and absorbance was recorded at 450 nm.

### 4.11. Statistics

All data are presented as the mean ± standard error of the mean (SEM) (*n* = 6). Statistical analyses were performed using GraphPad Prism version 5, and differences between groups were determined by one-way analysis of variance (ANOVA) followed by Tukey’s post hoc test. For repeated measurements, we analyzed the AHR data using Two-way repeated measures ANOVA followed by Bonferroni’s post hoc test to account for the repeated methacholine measurements within the same animals. Differences were considered statistically significant at *p* < 0.05. Significance levels are indicated as follows: *** *p* < 0.001, ** *p* < 0.01, and * *p* < 0.05 compared with the vehicle-treated group; ^###^ *p* < 0.001, ^##^ *p* < 0.01, and ^#^ *p* < 0.05 compared with the OVA-treated group.

## Figures and Tables

**Table 1:** Primer sequence.

Gene	Primer Sequence
*Il-4*	sense 5′-TCA ACC CCC AGC TAG TTG TC-3′, antisense 5′-TGT TCT TCG TTG CTG TGA GG-3′
*Il-5*	sense 5′-CAC CAG CTA TGC ATT GGA GA-3′, antisense 5′-CTC TTT TTG GCG GTC AAT GT-3′
*Il-13*	sense 5′-CTG GAT TCC CTG ACC AAC AT-3′, antisense 5′-GGT TAC AGA GGC CAT GCA AT-3′
*Inf-* *γ*	sense 5′-CAC GGC ACA GTC ATT GAA AG-3′, antisense 5′-GTC ACC ATC CTT TTG CCA GT-3′
*Il-17A*	sense 5′-AGC TGG ACC ACC ACA TGA AT-3′, antisense 5′-AGC ATC TTC TCG ACC CTG AA-3′
*Gapdh*	sense 5′-AAC TTT GGC ATT GTG GAA GG-3′, antisense 5′-GGA TGC AGG GAT GAT GTT CT-3′

## Data Availability

The original contributions presented in this study are included in the article/Supplementary Materials. Further inquiries can be directed to the corresponding author.

## Supplementary Materials

The following supporting information can be downloaded at https://www.mdpi.com/article/10.3390/ijms27167296/s1.

## Abbreviations

LPA_5_, lysophosphatidic acid receptor 5; AS2717638, 6,7-dimethoxy-2-(5-methyl-1,2-benzisoxazol-3-yl)-4-(1-piperidinylcarbonyl)-1(2H)-isoquinolinone; OVA, ovalbumin; IL-4, interleukin-4; IL-5, interleukin-5; IL-13, interleukin-13; IL-17A, interleukin-17A; IFN-γ, interferon-g; BALF, bronchoalveolar lavage fluid; PAS, Periodic acid–Schiff; ELISA, enzyme-linked immunosorbent assay; PBS, phosphate-buffered saline; PCR, polymerase chain reaction; HRP, horseradish peroxidase; GAPDH, glyceraldehyde-3-phosphate dehydrogenase; H&E, hematoxylin and eosin; AHR, airway hyperresponsiveness; DEX, dexamethasone; DNP, dinitrophenyl; HSA, human serum albumin.
