# Supplementary material for: Lysophosphatidic Acid Receptor 5 (LPA5) Antagonist AS2717638 Attenuates Allergic Asthma by Reducing Mast Cell Degranulation and Th2 Inflammation in Mice
Source: Int J Mol Sci. 2026 Aug 15;27(16):7296. doi: 10.3390/ijms27167296 (PMC13513380; doi:10.3390/ijms27167296)
Supplement: Supplementary file 1 [file ijms-27-07296-s001.zip › ijms-4440897-supplementary.pdf]

Supplemental Figure S1

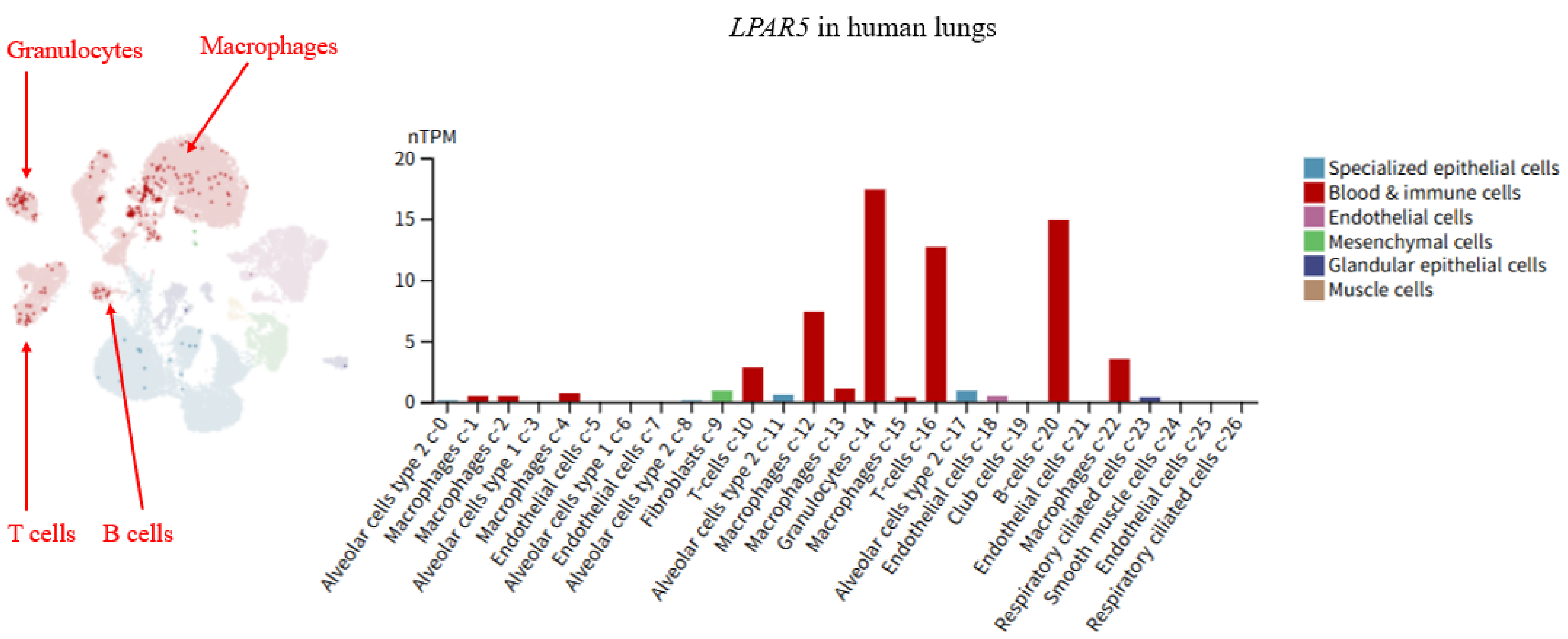

Supplemental Figure S1. Expression levels of *LPAR5* in various cell types of human lungs. Data is adopted from Human Protein Atlas website, which provides integrated data deposited by various researchers. The same data is available at https://v22.proteinatlas.org/ENSG00000184574-LPAR5).

Supplemental Figure S2

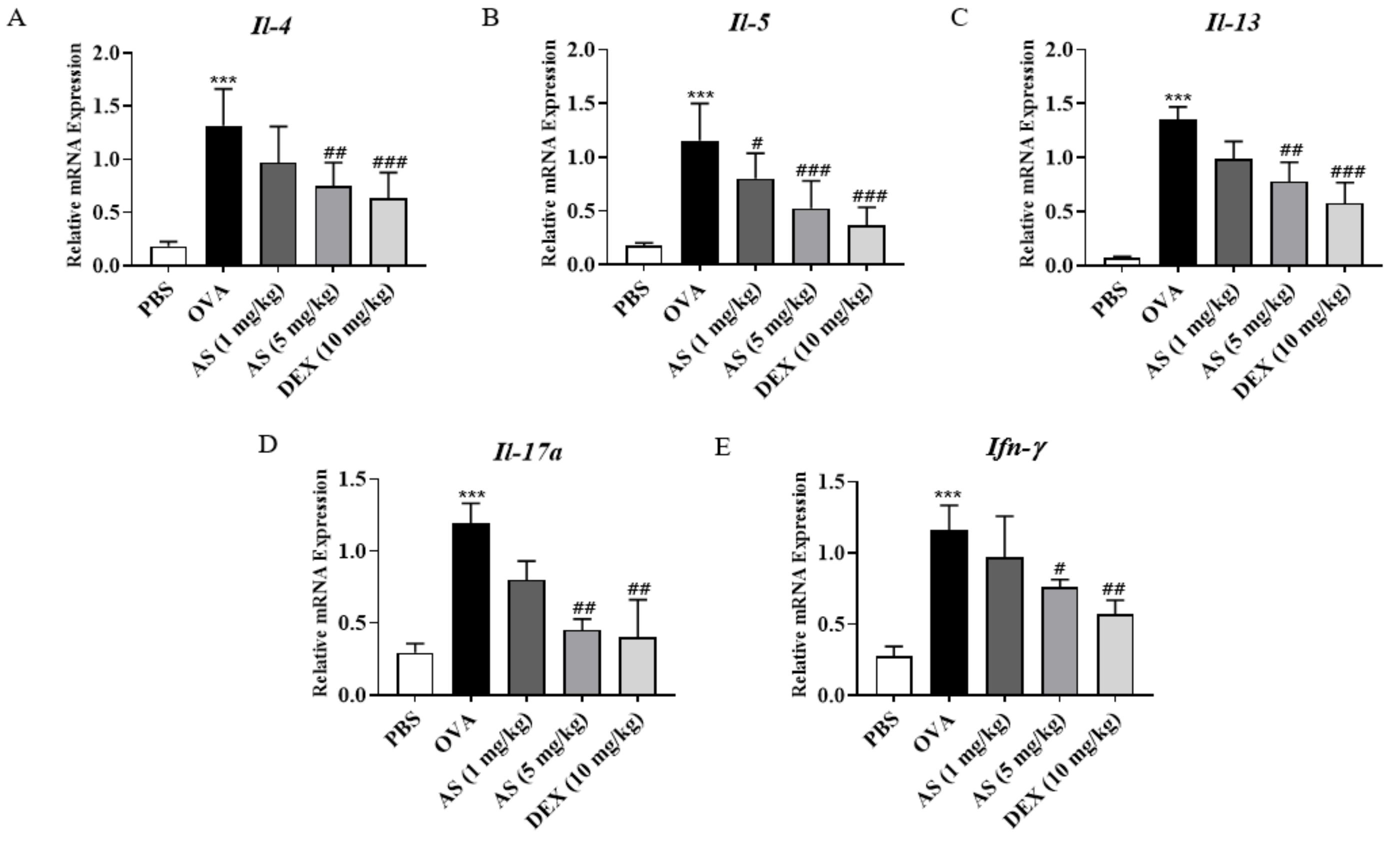

Supplemental Figure S2. AS2717638 inhibited the mRNA expression levels of Th2/Th17/Th1 cytokine expression in the lung tissues. (A-E) mRNA expression of Th2 cytokines, *Il-4* (A), *Il-5* (B), and *Il-13* (C), Th17 cytokine, *Il-17A* (D), Th1 cytokine, *Ifn-γ* (E), in the lung tissues. Levels of cytokine mRNAs are presented as normalized ratios relative to the mRNA level of *Gapdh*. The results are presented as the mean ± SE values of qPCR (n = 6). $^{**}p < 0.01$, $^{***}p < 0.001$ *vs.* the PBS-treated group, $^{\#}p < 0.05$, $^{\#\#}p < 0.01$, $^{\#\#\#}p < 0.001$ *vs.* the OVA-treated group.
